# *Blastocystis* subtyping and its association with intestinal parasites in children from different geographical regions of Colombia

**DOI:** 10.1371/journal.pone.0172586

**Published:** 2017-02-21

**Authors:** Juan David Ramírez, Carolina Flórez, Mario Olivera, María Consuelo Bernal, Julio Cesar Giraldo

**Affiliations:** 1 Grupo de Investigaciones Microbiológicas – UR (GIMUR), Programa de Biología, Facultad de Ciencias Naturales y Matemáticas, Universidad del Rosario, Bogotá – Colombia; 2 Grupo de Parasitología, Instituto Nacional de Salud, Bogotá – Colombia; 3 Programa profesional de Biología, Grupo de Investigación en Parasitología y Microbiología tropical – GIPAMT, Universidad Incca de Colombia, Bogotá – Colombia; Charles University, CZECH REPUBLIC

## Abstract

*Blastocystis* is a common enteric protist colonizing probably more than 1 billion people with a large variety of non-human hosts. Remarkable genetic diversity has been observed, leading to the subdivision of the genus into multiple subtypes (ST), some of which are exclusively found in non-human hosts. The aim of this study was to determine the distribution of *Blastocystis* STs/18S alleles in symptomatic (abdominal pain, anal pruritus, diarrhea, headache, nauseas and/or vomit) and asymptomatic children from nine geographical regions of Colombia. A total of 2026 fecal samples were collected as part of a national survey to estimate the frequency of intestinal parasites in children. A set of 256 samples that were *Blastocystis* positive was finally selected. The samples were submitted to DNA extraction, Real Time PCR and sequencing using *Blastocystis*-specific primers targeting the small subunit rRNA gene for ST identification. DNA of *Ascaris lumbricoides* (16.4%), *Trichuris trichiura* (8.2%), hookworms (*Necator americanus/Ancylostoma duodenale*) (7.3%), *Giardia duodenalis* (23.1%), *Entamoeba* complex (82%), *Entamoeba coli* (55%), *Hymenolepis nana* (0.8%), *Endolimax nana* (33.2%) and *Neobalantidium coli* (2.7%) was detected in the *Blastocystis-*positive samples. We detected ST1 (21.4%), ST2 (19.5%), ST3 (55.5%), ST4 (0.8%), ST6 (2%) and ST7 (0.8%); alleles 1, 2, 4, 81, 82 and 83 for ST1; alleles 9, 11, 12, 15, 67, 71 and 73 for ST2; alleles 34, 36, 38, 45, 49, 55, 134 and 128 for ST3; allele 42 for ST4; allele 122 for ST6, and allele 142 for ST7. Further studies implementing high-resolution molecular markers are necessary to understand the dynamics of *Blastocystis* transmission and the role of this Stramenopila in health and disease.

## Introduction

*Blastocystis* is a single-celled Stramenopila protist that colonizes probably around 1 billion people and presents a number of genetic variants living in the gastrointestinal tract of humans, farm animals, birds, rodents, reptiles and others [1,2]. Some authors have associated its presence to abdominal pain, constipation, diarrhea, flatulence and irritable bowel syndrome-like symptoms (IBS) [3,4] and even to skin disorders as urticaria [5]. The transmission occurs via fecal-oral route; the extent to which human-human, human-animal and animal-human transmission occurs remains under debate [1,6–9].

One of the major drivers of *Blastocystis* ubiquity is its genetic diversity. A set of several distinct ribosomal lineages called subtypes (ST), have been reported. In 2007, a consensus of *Blastocystis* nomenclature was proposed revealing nine distinct STs colonizing humans, other mammals and birds, based on polymorphic regions across the small subunit (SSU) rRNA gene and sequence tagged sites [10]. So far, no strict associations between the STs and the hosts have been reported, although moderate host specificity is seen [4,11]. Today, at least 17 genetically distinct SSU rRNA clusters are known [11]. Furthermore, genetic diversity studies using Multilocus Sequence Typing (MLST) schemes have shown that intra-genetic diversity varies dramatically among the STs [9].

Moderate host specifity is seen regarding *Blastocystis* subtypes. Humans are generally infected with nine STs where STs 1, 2, 3 and 4 are the most frequent. Albeit, ST1, ST2 and ST3 seem to be equally prevalent in patients with diarrhea and healthy individuals; and ST4 appears to be linked to diarrhea and/or irritable bowel syndrome-like (IBS) in Europe [11–13]. Molecular epidemiology studies have demonstrated that humans can be colonized by one or more of several STs, some of which are commonly found in non-human hosts, which highlights a plausible zoonotic potential. The information in Latin America is scarce and studies in Colombia, Brazil, Argentina and Mexico have shown that humans are mainly infected with STs 1, 2 and 3 [5,14–16]. While these studies demonstrate the absence of ST4 in human’s native to Latin America, other reminiscent studies showed the presence of ST4 in non-human primates in Colombia at low proportions [14,16]. A recent study from South-America showed the presence of ST4 in humans [14]. Curiously, in Colombia, there was observed a strict association between STs and clinical phenotypes and in Argentina, *Blastocystis* infection was associated to urticaria implying the need to understand the clinical relevance of *Blastocystis* infection in this region of the world.

Therefore, the aim of this study was to conduct a national survey in Colombia to obtain *Blastocystis* positive stool specimens and develop SSU rRNA *Blastocystis* barcoding. We established the geographical distribution of *Blastocystis* STs in symptomatic and asymptomatic children from Colombia.

## Materials and methods

### Ethical statement

**Fig 1:**
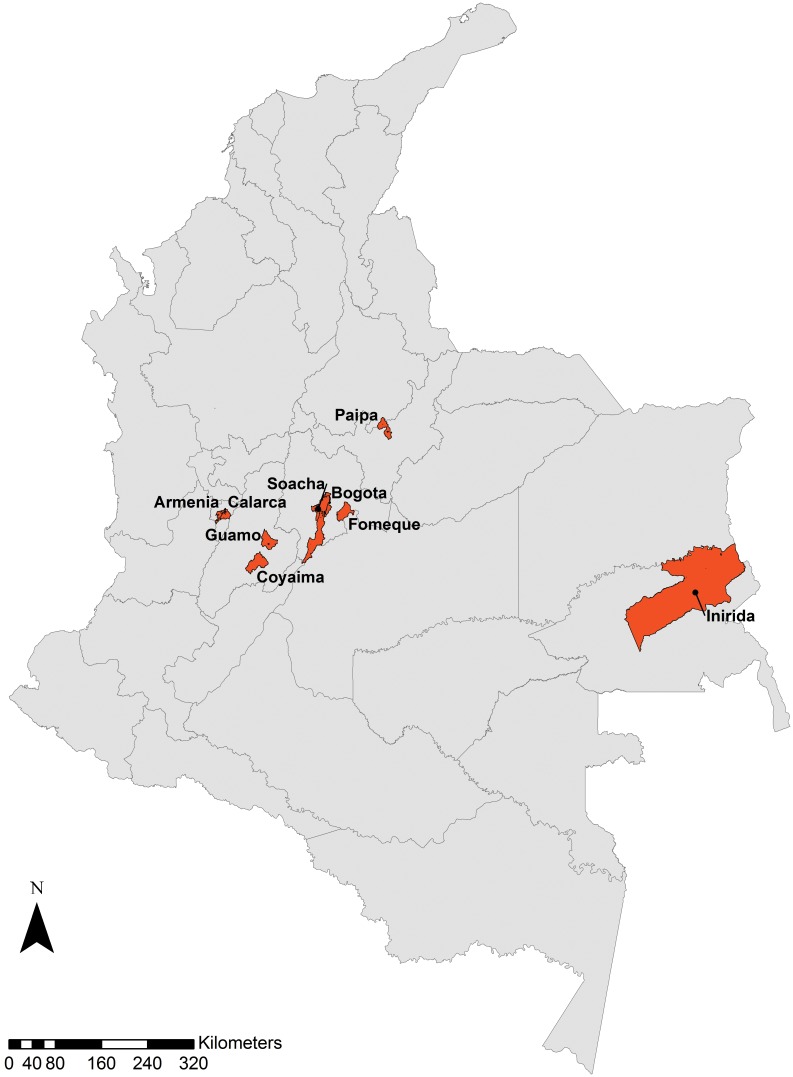
Geographical location of the nine localities where stool samples from children were collected.

Fecal samples were collected from nine locations across Colombia from symptomatic (diarrhea, abdominal pain, headache, anal pruritus, vomit and/or nauseas) and asymptomatic children ranging from 5–14 years old from 2012 to 2013. The status of symptomatic and asymptomatic children was established by a survey developed by a physician in each area. A parent or guardian of any child participant of this study provided written informed consent on their behalf. The ethical clearance of this study was followed by the ethics of Helsinki declaration and resolution No. 008430 of 1993 from the Ministry of Health from Colombia and “El Código del Menor”. The study protocol was approved by the ethics committee from Universidad INCCA de Colombia under the Number 237894. A total of 2026 samples were collected as part of a national study that aimed to establish the frequency of intestinal parasites in Colombian children. The localities sampled were Puerto Inirida (Rural, 249 samples), Guamo (Rural, 222 samples), Coyaima (Rural, 213 samples), Calarcá (Peri-Urban, 178 samples), Armenia (Urban, 333 samples), Bogotá (Urban, 275 samples), Soacha (Urban, 200 samples), Fomeque (Urban, 108 samples) and Paipa (Urban, 248 samples) (Fig 1). This is not a study that intended to estimate prevalence and the size sample was by statistical convenience (no size calculation due to the lack of information of prevalence of intestinal parasites in the areas selected and we recruited the patient that voluntary provided a fecal sample to the study). GMO permits are not needed according to the local legislation.

### Study population, microscopy and DNA extraction

For this study, we selected all samples that were *Blastocystis* positive by microscopy. A total of 256 samples were selected from Puerto Inirida (Rural, 14 samples), Guamo (Rural, 36 samples), Coyaima (Rural, 30 samples), Calarcá (Peri-Urban, 24 samples), Armenia (Urban, 30 samples), Bogotá (Urban, 31 samples), Soacha (Urban, 26 samples), Fomeque (Urban, 20 samples) and Paipa (Urban, 45 samples). The overall *Blastocystis* frequency of infection across the population studied was 12.6% (256/2026). In total, 140 males and 86 females that ranged from 5–14 years old were identified as positive. The fecal samples were collected in plastic recipients, labeled and conserved in refrigerated boxes. The samples were divided in two parts: one part was fixed in a proportion (1:4) in ethanol 70% and stored at -20°C for DNA extraction. The other part was used for conducting diagnosis of intestinal parasites including Lugol's stain and trichrome staining to detect *Blastocystis*. DNA was extracted from 200mg of stool using the MP FastDNA for Soil kit (MP Biochemicals, Solon, OH) according to the manufacturer´s instructions and the modifications reported elsewhere [17, 18].

### Molecular diagnosis of *Blastocystis* and intestinal parasites

We conducted Real Time PCR for the detection of *Blastocystis* and intestinal parasites DNA using primers and Taq-man probe species-specific as reported elsewhere in the 256 samples (*Blastocystis-*positive) [17,19]. Assays were conducted in 96-well MicroAmp optical plates (Applied Biosystems, Foster City, California, USA). All reactions were conducted in duplicate and in a total volume of 20μL containing 7μL of Taqman Fast Master Mix (Applied Biosystems), 2 μL of species-specific primers (10 μM), 3 μL of Taqman probes (5 μM), 4 μL of DNA template and water to give a final volume of 20 μL. The PCR mixtures were run on an ABI7500 Fast Real-Time PCR system (Applied Biosystems). Therefore, we conducted experiments to establish the dynamic range of our assay using standards from 10,000fg/ μL to 1fg/μL. For quantification, plasmids containing the target sequences were cloned into the pGEM–T Easy Vector System I (Promega, Madison, USA), according to the manufacturer’s instructions, and transformed into XL1-Blue *Escherichia coli* (Agilent Technologies, UK). The transformed colonies containing the plasmids were extracted by using the QIAprep Spin Miniprep Kit (QIAGEN, Valencia, CA). The purified plasmid DNA was quantified by using a Nanodrop and diluted to have a concentration range of 10000fg/ μL to 1fg/μL. These were the standards to quantify the unknown samples in an absolute Real-Time PCR scheme. Positive DNA from each parasite and negative controls were always included in each run. We were able to perform the PCRs in order to identify the eight parasites cited in ref. [17] only and none others. We analyzed the dynamic range to determine a Ct cut-off value to determine positive and negative samples for intestinal parasites. In overall with the intercept of the normalized data we concluded that the Ct Cut-off for our assay was 37.6.

### Discrimination of *Blastocystis* STs

A total of 256 samples were submitted to subtyping via PCR amplification of *Blastocystis*-specific SSU rDNA using the primers RD5 (5´-ATC TGG TTG ATC CTG CCAG T-3´) and BhRDr (5´-GAG CTT TTT AAC TGC AAC AAC G- 3´) [20], as recently recommended [21]. PCR products were purified and sequenced by both strands using de dideoxy-terminal method using the Applied Biosystems^®^ 310 Genetic Analyzer. Sequences were edited in MEGA 4.0 [22] and compared with retrieved reference sequences from representing each ST in GenBank by BLAST queries. Additionally, sequences were submitted to sequence queries at the *Blastocystis* 18S database available at http://pubmlst.org/blastocystis/ for *Blastocystis* 18S allele calling and confirmation of ST. The resulting sequences were deposited on GenBank under the accession numbers KX963577-KX963768.

### Statistical analyses

Associations between frequency of infection and sex or age groups were assessed by Pearson X^2^ test or Fisher's exact test. Bivariate and multivariate logistic regression was used to estimate the association between demographic, socioeconomic variables, location and other parasites. A value of p <0.05 was considered statistically significant. Statistical analysis was performed using STATA version 10.1 (Stata Corp, College Station, TX).

## Results

### Symptoms and intestinal parasites frequency in a cohort with *Blastocystis* infection

The population of children selected showed abdominal pain (10.5%), anal pruritus (2%), diarrhea (2%), headaches (2.7%), nauseas (8.6%), vomit (2.3) and 71.9% of the patients surveyed were asymptomatic. We detected *A*. *lumbricoides* (16.4%), *T*. *trichiura* (8.2%), hookworms (7.3%), *G*. *duodenalis* (23.1%), *Entamoeba* complex (82%), *E*. *coli* (55%), *Hymenolepis nana* (0.8%), *Endolimax nana* (33.2%) and *Neobalantidium coli* (2.7%).

### *Blastocystis* subtypes and 18S alleles

**Fig 2:**
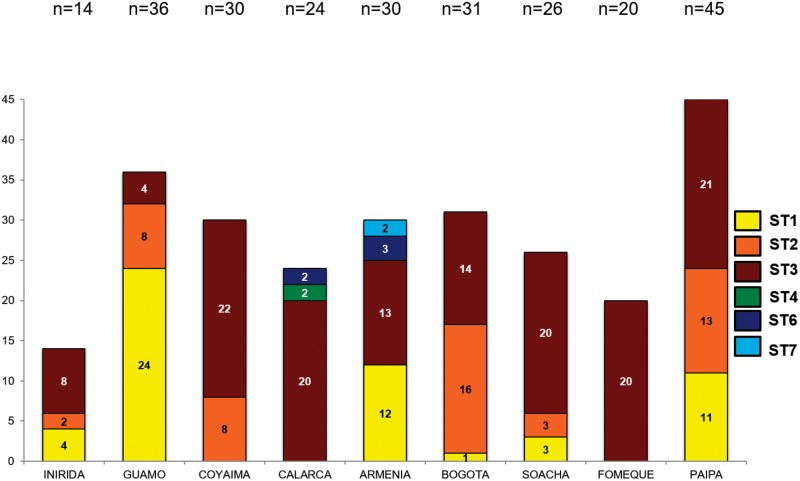
Frequency and distribution of *Blastocystis* STs detected in the nine localities analyzed.

**Fig 3:**
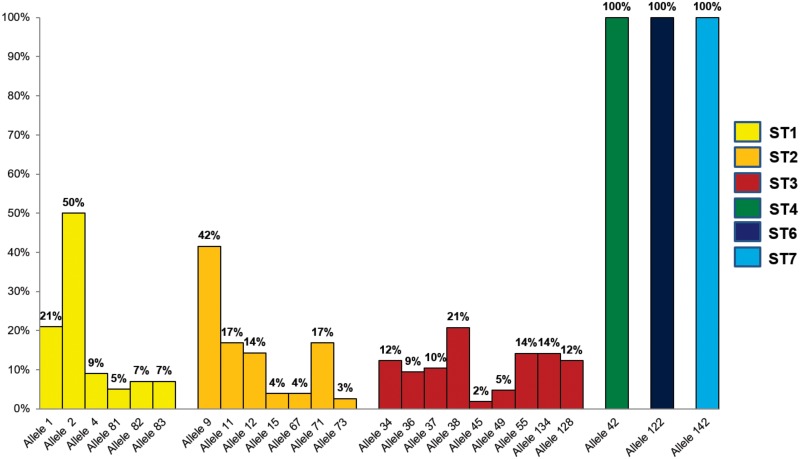
Frequency and distribution of *Blastocystis* 18S alleles detected in the 256 stool samples analyzed.

DNA barcoding targeting the SSU rDNA region was performed. STs amplified belonged to ST1 (21.4%), ST2 (19.5%), ST3 (55.5%), ST4 (0.78%), ST6 (1.9%) and ST7 (0.78%) (Fig 2). Also, 14 samples showed mixed infections with STs 1 and 3. Furthermore, we retrieved the 18S alleles for each subtype observing alleles 1, 2, 4, 81, 82 and 83 for ST1; alleles 9, 12, 15, 67, 71 and 73 for ST2; alleles 34, 36, 37 38, 45, 49, 55, 134 and 128 for ST3; allele 42 for ST4; allele 122 for ST6 and allele 142 for ST7 (Fig 3). STs 1, 2 and 3 showed the highest DNA concentration in fg/uL but there were no statistically significant differences among DNA load and STs.

### Statistical associations

**Fig 4:**
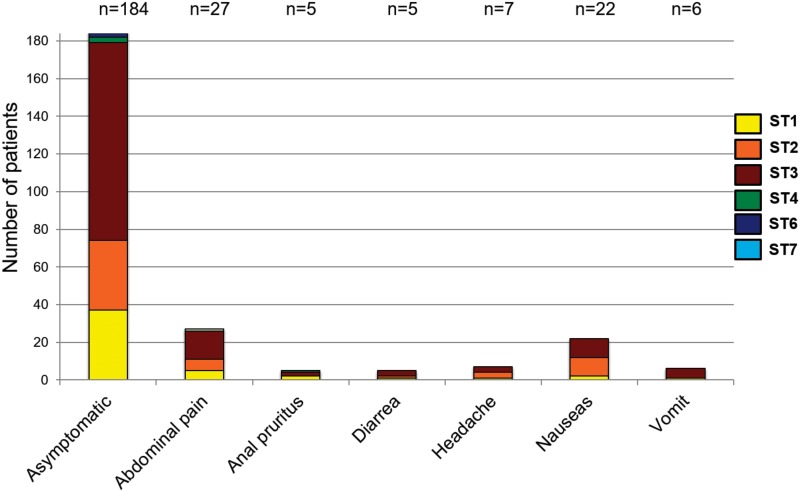
Distribution of symptoms according to each *Blastocystis* ST.

**Fig 5:**
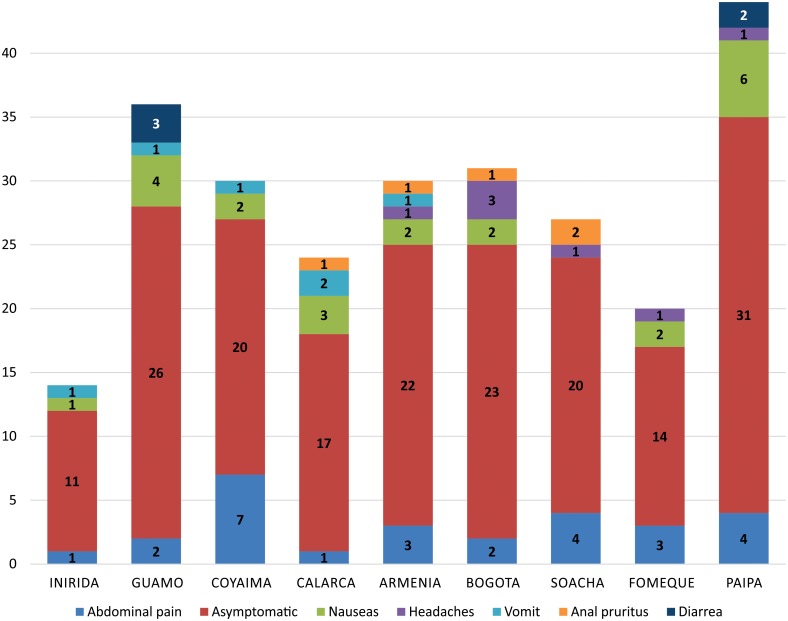
Frequency of asymptomatic and symptomatic patients by geographical region.

Statistical analyses were conducted to determine if there were any associations between parasite infections, age and sex across *Blastocystis*-infected individuals (Table 1). Only co-infection with *E*. *nana* was significant. The other parasites did not show statistical significant associations between these variables (sex and age) and parasitic infection. When univariate and bivariate analyses were conducted in order to determine if there were associations among sociodemographic stratification and location, we detected no statistical significance. We only observed statistical significance in hookworms with *G*. *duodenalis* (OR = 3.13 CI = 1.70–5.76, p<0.001) as shown in Table 1. There was no statistical association between *Blastocystis* infection and/or STs, and variables as sex, age, symptomatology or sociodemographic stratification and location. The distribution of *Blastocystis* STs and symptoms are shown in Fig 4. The frequency of symptoms by geographical location are shown in Fig 5.

**Table 1:** Total frequency and statistical associations by sex and age of intestinal parasites across 256 *Blastocystis*-infected individuals from nine locations of Colombia.

Parasites	Frequency			Sex			Age (Years)		
	% (n)	CI 95%	Pathogenic	Female	Male	P	5–9	10–14	P
**Helminths**									
*Ascaris lumbricoides*	16.4 (42)	12.1–21.5	Yes	39.0	61.0	0.184	80.5	19.5	0.730
*Hymenolepis nana*	0.78 (2)	0–2.7	Yes	50.0	50.0	1.000	50.0	50.0	0.373
*Trichuris trichiura*	8.2 (21)	5.1–12.2	Yes	28.1	71.9	0.339	78.9	21.1	0.955
Hookworms	7.3 (18)	4.2–10.8	Yes	20.0	80.0	0.668	60.0	40	0.285
**Protozoa**									
*Balantidium coli*	2.7 (7)	1.1–5.5	Yes	0	100	0.058	71.4	28.6	0.638
*Endolimax nana*	33.2 (85)	27.5–39.3	No	44.1	55.9	0.011	79.8	20.2	0.880
*Entamoeba coli*	55.1 (141)	48.8–61.3	No	32.1	67.9	0.656	77.1	22.9	0.368
*Entamoeba histolytica/E*. *dispar/E*.*moshkovskii*	82.0 (210)	76.8–86.5	Yes /No	32.5	67.5	0.565	79.9	20.1	0.563
*Giardia duodenalis*	39.8 (102)	33.8–46.1	Yes	30.7	69.3	0.469	75.3	24.7	0.206

## Discussion

*Blastocystis* is a recurrent commensal of the intestinal gut and its true pathogenic potential is still under investigation. Due to the epidemiological characteristics of South-America (lack of sanitation, lack of potable water systems and poverty) the frequency of infection tends to be high [16,23,24]. The information regarding the molecular epidemiology of *Blastocystis* subtypes in South-America is scarce with limited information from Colombia, Brazil and Argentina. Our results of frequency of infection across the whole population were pragmatic observing around 12% of infection in Colombian children, likely suggesting that the dynamics of transmission of this Stramenopila show interesting features in different geographical regions of Colombia (Table 1). These patients were also infected with other intestinal parasites as *Ascaris lumbricoides*, *Trichuris trichiura*, hookworms, *Entamoeba spp*. and *Giardia duodenalis* suggesting a high rate of intestinal parasites transmission implying the lack of sanitation and inadequate procedures of feces elimination in the localities studied (Table 1).

A total of nine geographical regions across the country were sampled, these regions show interesting features in terms of sociodemographic differences (rural and urban localities). However, this was not a predictor of *Blastocystis* infection highlighting the ubiquitous distribution of this protist regardless the sociodemographic profile of the patients (Table 1). When we undertook statistical analyses, we did not observe statistical significant associations between being infected by *Blastocystis* and/or other intestinal parasites, symptomatology, age, sex and sociodemographic characteristics (Table 1; Fig 4). Also, due to the pathogenic potential of each parasite, we cannot rule out that the symptomatology is strictly a consequence of *Blastocystis* colonization. There was one only statistical association when the bivariate analysis was conducted and was in the case of hookworms with *G*. *duodenalis* (OR = 3.13 CI = 1.70–5.76, p<0.001). These results suggest that these populations are polyparasited and governmental interventions are urgently required including molecular efforts to understand transmission profiles of *Giardia* as we have done here for *Blastocystis*.

A total of six STs were detected in the children surveyed (Fig 2). STs 1, 2 and 3 were the most frequent but particularly ST3 was the abundant across the dataset including 14 samples that showed mixed infections with STs 1 and 3. Previous studies in Colombia have shown that humans harbor STs 1, 2 and 3 with strict association to clinical phenotypes as asymptomatic, diarrhea and IBS respectively [16]. However, we did not find any statistical associations between symptomatic and asymptomatic patients and the fact of being infected by a particular ST (Figs 4 and 5). These clashing results might be explained by the difference in the population herein studied and that evaluated in [16]. Also, the samples tested in [16] were low compared to this study. We intriguingly observed that for example asymptomatic patients were infected by all the STs detected and particularly the ST6 was only observed in this group of patients. In the group of symptomatic patients, apart from STs 1, 2 and 3 that were the most abundant we observed ST4 in patients with anal pruritus and ST7 in patients with abdominal pain (Fig 4). One bias of our study is that the final selection of positive *Blastocystis* samples was conducted with microscopy results. Some authors have shown that PCR is more sensitive than microscopy and might in fact impact the STs distribution. However, we randomly selected 30 samples that were negative by microscopy and just 1 sample turned out positive by PCR showing that our microscopy was sensitive enough for the final selection. This study had the opportunity to make a comment on the sensitivity of the microscopy technique vs. the molecular technique to detect *Blastocystis* infection from human fecal samples. The frequency of detection by microscopy was 12.6% (n = 256/2026). To confirm this, DNA was extracted from the 256 microscopy positive samples, plus 30 microscopy negative samples. This study determined that all 256 microscopy positive samples were PCR positive (no false positives), but it identified 1 PCR positive out of 30 microscopy negative samples, suggesting a false negative rate of 3.3%, indicating that the PCR technique is more sensitive, confirming previous results. Furthermore, that the estimated prevalence by PCR would thus be n = 315/2026 or 15.5% given a 3.3% False Negative rate. We are aware about the limitation of our study but at the same time we know that the most of the big dataset was diagnosed and subtyped. A bigger subset must be sampled to corroborate the values of sensitivity.

We detected new STs in Colombian patients as STs 6 and 7 (Fig 1). It is quite intriguing that these new STs (6 and 7) were detected, particularly in rural areas as Calarca and urban as Armenia. These subtypes have been previously reported in Africa, Central Asia and Australia [3,11,25–27]. In Europe, STs 2, 3 and 4 are frequent in human infections; in this geographical scenario ST3 is the most abundant in human patients [20,28]. ST6 has been frequently reported in birds from Colombia and described in *Passer domesticus*, *Oryzoborus maximiliani*, *Sicalis flaveola* and *Petrochelidon pyrrhonota* [16]. Also ST6 was reported in humans and associated to IBS in different countries as France, Greece, Egypt and Nepal [19,29–31]. In our dataset, the five patients (1.9%) turned out to be asymptomatic contrasting findings around the globe. Finally, ST7 was also detected; this ST has been reported in Denmark, Egypt, Nepal, Australia and Iran and also related to IBS [30,32,33]. The STs detected in the Colombian population of symptomatic and asymptomatic children suggest a high degree of zoonotic transmission. It is necessary to sample animals close to the human populations to prove this hypothesis. A series of alleles were detected in our samples, some of the alleles had already been reported for Colombia but others were brand new (Fig 3). STs 1, 2 and 3 showed the highest frequency of alleles (22 in total). In the case of samples typed as ST4, we detected allele 42 which is frequently detected in Europe [4]. For ST6 the allele detected was 122 and previously reported in Colombian birds (Fig 3).

Regarding the frequency of infection by other intestinal parasites. It is clear that the incidence of intestinal parasites is higher in children than in adults because of the lack of natural resistance and differences in their behavior and habits. The above closely related to the environmental determinants and socio-economic characteristics of a population, which generate a higher risk of infection. The World Health Organization (WHO) in its action plan raises early protection of children and vulnerable communities; detection, treatment and control of intestinal parasites are subject to follow, because this is one of the leading causes of morbidity and mortality in children worldwide [34]. The object then is to improve and/or strengthen the diagnosis of parasitic infections, for which the use of molecular tools arises due to its high sensitivity allowing specific identification of various pathogens, i.e. species identification and subpopulations as is the case of *Giardia* assemblages and *Blastocystis* STs. This individual level translates into specific and timely treatment to the organism and better clinical management of the patients. For example, recent reports have shown that giardiasis in childhood is associated with malnutrition, stunting and impaired cognitive development, regardless of the presence of diarrhea, prevalent among children two to five years old, according to several reports from developing countries [35].

An immune response, which can be individually variable and influenced by nutritional status, genetic factors, and repeated exposure to *G*. *intestinalis* suggesting that, contributes to low detection rates of *Giardia* seen in older children as well as an explanation to the asymptomatic infections [36]. Our results are in accordance with these premises, where we observed high rates of infection for *Giardia* and also for other parasites such as *Entamoeba*, *Ascaris*, *Trichuris* and hookworms. Also, the frequencies of infection for *Giardia* are in accordance to other reports in Colombia [37]. The differences are striking in terms of the sensitivity of the techniques which are also supporting the fact of using molecular methods instead of conventional microscopy as has been clearly stated by different authors [38–40]. These can be explained by the intermittent excretion of cysts in *Giardia* and also the expertise of the operator who implements microscopy.

In conclusion, we detected STs 1, 2 and 3 as the most frequent with the foreseen evidence of STs 4, 6, 7 with new alleles circulating in the country. We also observed that asymptomatic patients can harbor any *Blastocystis* ST suggesting no strict association between ST and symptoms. These results highlight the importance of *Blastocystis* infection in developing countries and the need to pursue studies using molecular epidemiology strategies to untangle the transmission dynamics of this protozoan with the aim of establishing intervention strategies that mitigate intestinal parasites transmission. New molecular markers need to be developed for a better understanding of *Blastocystis* diversity to deploy robust hypothesis about transmission dynamics and evolution of this Stramenopila.
